# Iridovirus CARD Protein Inhibits Apoptosis through Intrinsic and Extrinsic Pathways

**DOI:** 10.1371/journal.pone.0129071

**Published:** 2015-06-05

**Authors:** Chien-Wen Chen, Ming-Shan Wu, Yi-Jen Huang, Pei-Wen Lin, Chueh-Ju Shih, Fu-Pang Lin, Chi-Yao Chang

**Affiliations:** 1 Molecular Genetics Laboratory, Institute of Cellular and Organismic Biology, Academia Sinica, Taipei, Taiwan; 2 Institute of Fisheries Science, National Taiwan University, Taipei, Taiwan; 3 Institute of Bioscience and Biotechnology, National Taiwan Ocean University, Keelung, Taiwan; IISER-TVM, INDIA

## Abstract

Grouper iridovirus (GIV) belongs to the genus *Ranavirus* of the family *Iridoviridae*; the genomes of such viruses contain an anti-apoptotic caspase recruitment domain (CARD) gene. The GIV-CARD gene encodes a protein of 91 amino acids with a molecular mass of 10,505 Daltons, and shows high similarity to other viral CARD genes and human ICEBERG. In this study, we used Northern blot to demonstrate that GIV-CARD transcription begins at 4 h post-infection; furthermore, we report that its transcription is completely inhibited by cycloheximide but not by aphidicolin, indicating that GIV-CARD is an early gene. GIV-CARD-EGFP and GIV-CARD-FLAG recombinant proteins were observed to translocate from the cytoplasm into the nucleus, but no obvious nuclear localization sequence was observed within GIV-CARD. RNA interference-mediated knockdown of GIV-CARD in GK cells infected with GIV inhibited expression of GIV-CARD and five other viral genes during the early stages of infection, and also reduced GIV infection ability. Immunostaining was performed to show that apoptosis was effectively inhibited in cells expressing GIV-CARD. HeLa cells irradiated with UV or treated with anti-Fas antibody will undergo apoptosis through the intrinsic and extrinsic pathways, respectively. However, over-expression of recombinant GIV-CARD protein in HeLa cells inhibited apoptosis induced by mitochondrial and death receptor signaling. Finally, we report that expression of GIV-CARD in HeLa cells significantly reduced the activities of caspase-8 and -9 following apoptosis triggered by anti-Fas antibody. Taken together, these results demonstrate that GIV-CARD inhibits apoptosis through both intrinsic and extrinsic pathways.

## Introduction

Apoptosis is a conserved, highly-regulated process that plays a vital role in development, tissue homeostasis, and the removal of damaged and virus-infected cells. The caspase recruitment domain (CARD) family of cellular proteins plays a vital regulatory role in apoptosis. CARD was first defined by Hofmann *et al*. [1], who found that the apoptotic signal was frequently mediated by the assembly of proteins containing homology domains of the same class, such as the death domain (DD) or death effector domain (DED) [2]. CARD domains were originally identified in apoptotic signaling proteins of 90 amino acid residues, which contain six α-helices; these proteins include four human aspartic acid-specific cysteine proteases (caspase-1, caspase-2, caspase-4, and caspase-9) [3, 4]; two human cellular homologues of viral apoptosis inhibitor IAP (c-IAP1 and c-IAP2) [5]; two *C*. *elegans* cell death proteins (Ced-3 and Ced-4) [6]; one equine herpes virus 2 (EHV2) viral protein (E10) [7]; and one human TNF-R1 TRADD-RIP complex-containing protein (RAIDD) [8]. Based on the biological roles of proteins containing this domain, a network of interactions required to recruit caspases to apoptosis signaling-complexes has been predicted. CARD domain-containing proteins associate through CARD-CARD interactions, and this allows them to regulate apoptosis. In addition to promoting caspase activation, CARD proteins have been found to participate in NF-κB signaling pathways related to innate or adaptive immune responses, including the teleost inflammatory response [9].

Based on their general structures, CARD proteins have been divided into the following sub-families: (I) NBD-CARDs (such as Apaf-1), which contain a nucleotide-binding domain, and recruit caspase-9 to form an apoptosome complex through CARD-CARD interactions with procaspase-9 [10]; (II) coiled-coil-CARDs (such as CARD-9) that interact with Bcl10, which in turn promotes NF-κB activation upon T- and B-cell receptor stimulation [11]; (III) proteins containing CARD, bipartite-CARD, and protease domains (such as caspase-8 and caspase-9), which function as caspase signaling initiators; and (IV) CARD-only proteins (such as ICEBERG [12], INCA [13] and COP/Pseudo-ICE [14]), which act as non-enzymatic decoys that regulate caspase-1 activity in human.

During apoptotic signal transduction, caspase-8 and caspase-9 mediate the death receptor (extrinsic) and the mitochondrial (intrinsic) pathway, respectively. Activated caspase-8 and caspase-9 can recruit and cleave procaspase-3, the effector caspase; activated caspase-3 subsequently induces the irreversible apoptosis process [15]. Caspase-8 also cleaves the pro-apoptotic protein Bid, thereby activating the mitochondrial death pathway [16]. Bid bridges the intrinsic and extrinsic pathways, and can be used to amplify the apoptotic signal [17].

CARD-only proteins are found in many viruses; these include the CARD-like caspases of *Rana tigrina* ranavirus (UniPortKB: Q2WER7) [18], *Ambystoma tigrinum* stebbensi virus (UniPortKB: Q6YH84) [19], Soft-shelled turtle iridovirus (UniPortKB: C3RWR6) [20], Epizootic haematopoietic necrosis virus (UniPortKB: D3TTS4) [21], and CARD-containing protein 064R (UniPortKB: Q6GZR1) of Frog virus 3 [22]. To date, the functions of viral CARD-only proteins remain unknown.

Host cells utilize apoptosis as a primitive defense mechanism against viral infection. This innate host response can efficiently remove virus-infected cells, thereby limiting virus reproduction, and reducing or eliminating dissemination of progeny virus in the host [23]. However, several viruses have evolved strategies to counteract the death signaling machinery [24]. Large DNA viruses, such as adenoviruses [25], herpes viruses [26], and poxviruses [27], contain sequence homologs of Bcl-2, and herpes viruses [28] and molluscum contagiosum virus [29] contain v-FLIP (FLICE inhibitory protein), which has been shown to prevent apoptosis.

Grouper iridovirus (GIV) belongs to the *Ranavirus* genus of the *Iridoviridae* family. *Ranavirus* hosts include amphibians, fish, and reptiles. We have previously reported that UV-induced apoptosis is inhibited by (i) GIV infection of grouper kidney cells, and (ii) over-expression of GIV-Bcl (078R) in HeLa cells [30]. We also reported the presence of an open reading frame (ORF 027L) in the GIV genome encoding a CARD-only gene (GIV-CARD) [31]. This discovery prompted us to investigate whether GIV-CARD can inhibit apoptosis initiated by cell-like GIV-Bcl. We report our findings herein.

## Material and Methods

### Ethics statement

In this paper, we did not perform any animal research. However we used one viral stock (grouper iridovirus) which isolated from dead diseased yellow grouper of fish farm in southern Taiwan at 1999 July. We brought the fish sample back to lab with ice and isolated virus from spleen. In year 2000, we published grouper iridovirus in *Journal of Fish Diseases*. And we had cited reference [32] in this paper Material and Methods section. At that time Academia Sinica not yet set up IACUC, we did not have protocol for that isolation. After Academia Sinica IACUC set up (2003), our animal research all followed AS IACUC legislation. Our recent AS IACUC protocol ID: 11-10-229 is for the “Study of the interaction mechanism of orange-spotted grouper antiviral protein Mx against grouper iridovirus”.

### Virus and cells

Grouper iridovirus was isolated from diseased yellow grouper spleen tissue. Propagation and purification of GIV and isolation of the virus genome were performed as described previously [32, 33]. Grouper kidney (GK) [32] and HeLa cells were cultured in Leibovitz’s L15 or DMEM (Gibco) media supplemented with 10% heat-inactivated fetal bovine serum (FBS) (Gibco), L-glutamine (Gibco), and penicillin-streptomycin (Gibco) at 28 (GK) or 37°C (HeLa). L15 media supplemented with 2% FBS was used for viral infection.

### Construction of plasmids

GIV-CARD 027L ORF (Accession Number: AAV91050) [31] was amplified by polymerase chain reaction (PCR) from purified GIV genomic DNA for plasmid construction. The primers used for cloning are shown in Table 1. PCR was carried out under the following conditions: 5 min. at 94°C; 35 cycles of 30 s at 94°C, 30 s at 60°C, and 30 s at 72°C; and 7 min. at 72°C. The amplified fragments were digested with the relevant restriction enzymes, and then sub-cloned into vectors cleaved by the same enzymes. The inserted sequences were then amplified using the GIV-CARD-FLAG-F/GIV-CARD-FLAG-R or GIV-CARD-EGFP-F/GIV-CARD-EGFP-R primer pairs, and sub-cloned into pcDNA3CF [34] or pEGFP-N1 (Clontech) to generate plasmids pcDNA3CF_GIV-CARD or pEGFP-N1_GIV-CARD, respectively. The identities of these constructs were confirmed by restriction enzyme digestion and nucleotide sequence analysis.

**Table 1:** Primers used for polymerase chain reaction in this study.

Primer	Sequence
GIV-CARD-FLAG-F	CGCGGATCCGATGTACACTTCAAACTGTATGTTGAA
GIV-CARD-FLAG-R	CGGAATTCCTCAAGTTCCATCAAAACGGCGAGCTCT
GIV-CARD-EGFP-F	CGGAATTCCGATGTACACTTCAAACTGTATGTT
GIV-CARD-EGFP-R	CGCGGATCCGCCTCAAGTTCCATCAAAACGGCG
GIV-CARD-F	ATGTACACTTCAAACTGTATGTTGAAAC
GIV-CARD-R	CTACTCAAGTTCCATCAAAACGG
T7- GIV-CARD-F	TAATACGACTCACTATAGGATGTACACTTCAAACTGTATGTTGAAAC
T7- GIV-CARD-R	TAATACGACTCACTATAGGCTACTCAAGTTCCATCAAAACGG
GIV-CARD-5UTR-F	GATAGACGTTGGCTGTGCAATC
GIV-CARD-3UTR-R	GAACGCAATAGTTGCAAACCTTTTTTC
GIV-TNFR029L-F	ATGTTACAGACTGTTGTGATTTTG
GIV-TNFR029L-R	TTACAATATATTGAAGTAGGGGCG
GIV-TNFR030L-F	ATGCTGTTTGTAGCGCTCGTCTTG
GIV-TNFR030L-R	TTAATCCATTTTGCGGTTCTGTTG
GIV-TNFR065R-F	ATGTTAGTAGTAGGGATGTTG
GIV-TNFR065R-R	TTAAAACGCGCAAATTTTGTTTG
GIV-Bcl-F	ATGACGA ATATAAACTTTT
GIV-Bcl-R	TGGTAACAGATAATAAGCAATTACGGC
GIV-MCP-F	CATGAGGTTCTCGCACGC
GIV-MCP-R	CCCATGGAACCGTTCATG
Grouper-β-actin-F	GCCCCACCAGAGCGTAAATA
Grouper-β-actin-R	CATCGTACTCCTGCTTGCTGAT
qGIV-CARD-F	GGTGTGATAAATCGGGGAGA
qGIV-CARD-R	GCAAACCTT TTTTCCTACTCAAGT
qGIV-TNFR029L-F	CGTGCTCTGTCGGATACAAA
qGIV-TNFR029L-R	CAGTCCAATTTTGCTCGTGA
qGIV-TNFR030L-F	GTAGCGCTCGTCTTGATTCC
qGIV-TNFR030L-R	TCTGTCATCGAACGTGCATT
qGIV-TNFR065R-F	GGTAGCGTCTCACTGCTCCT
qGIV-TNFR065R-R	ATTTGCCACACACCCTGTCT
qGIV-Bcl-F	TCAAACGGAGGCTGGGTAAC
qGIV-Bcl-R	GTTCCAAACAAAGCGCCGAA
qGIV-MCP-F	CCAGCCTCACGTACGAAAAT
qGIV-MCP-R	TTACGGTGATGCYAGCGTTG

Restriction sites and T7 promoter sequences are underlined for the primer pairs used for cloning and *in vitro* transcription, respectively.

### Sequence analysis and structure modeling

Database similarity searches were performed using the National Center for Biotechnology Information (NCBI) BLAST server [35]. Sequence alignments were performed using the ClustalW2 (EMBL-EBI, http://www.ebi.ac.uk/) web service. A phylogenetic tree was constructed using MEGA 6 (Ver.6.0.5) software with a Neighbor-Joining Tree program. The GIV-CARD homology model was obtained using the crystal structure of human ICEBERG (PDB code: 1DGN) as template for the Automated Modeling tool of the Swiss-Model web service (http://swissmodel.expasy.org/) [36–38], and the structural model of GIV-CARD was presented using PyMOL (Ver.1.6) software. Helical regions were predicted based on the alignment data acquired through Automated Modeling.

### Northern blot hybridization

Total RNA was prepared using TRIzol reagent (Invitrogen) from GIV-infected GK cells at a multiplicity of infection (m.o.i.) of 10. Ten micrograms of RNA were separated on a 1% formaldehyde agarose gel, and then transferred onto a Hybond-N membrane (Amersham Biosciences). The membrane was hybridized at 42°C overnight with a [^32^P]dCTP-radiolabeled GIV-CARD DNA probe, which was synthesized using GIV-CARD-F/GIV-CARD-R primer pairs (Table 1). After hybridization, the membrane was washed with a solution containing 0.1% SDS and 0.1× SSC, and subsequently exposed to Biomax X-ray film (Kodak) for signal detection. Control RNA was collected from mock-infected GK cells. Indicated cultures were pretreated for 1 h before infection with cycloheximide (CHX, final concentration of 200 μg/ml; Calbiochem) or aphidicolin (APH, final concentration of 5 μg/ml; Calbiochem), to inhibit protein or DNA synthesis, respectively.

### Expression of GIV-CARD-EGFP and GIV-CARD-FLAG in Hela cells

HeLa cells were cultured in DMEM media supplemented with 10% FBS (density of 1.5 × 10^5^ cells per well in a six-well multidish (Nunc)) at 37°C overnight. Cells were transfected with pEGFP-N1_GIV-CARD or pcDNA3CF_GIV-CARD (2 μg DNA/well) using LipofectAMINE 2000 (Invitrogen), in accordance with the manufacturer’s instructions. Transfected cells were examined at the indicated times using a fluorescence microscope system (Axiovert 200M Zeiss/Photometrics CoolSnap HQ) or immunofluorescence staining. Cell nuclei were co-stained with DAPI (D1306, Invitrogen).

### Preparation of GIV-CARD dsRNA

To knockdown GIV-CARD expression, GIV-CARD double stranded RNA (dsRNA) was prepared *in vitro* in accordance with the T7 RiboMAX Express RNAi System Protocol (P1700, Promega). The T7 promoter sequence was added to gene specific primers, and GIV-CARD-F/T7-GIV-CARD-R and GIV-CARD-R/T7-GIV-CARD-F primer pairs (Table 1) were used to amplify sense and anti-sense DNA templates, respectively. The PCR product templates were purified using QIAquick PCR Purification Kits (28104, QIAGEN) following the manufacturer’s protocol. Purified PCR templates were quantified and used to synthesize single stranded RNA (ssRNA). Sense and anti-sense ssRNA were co-incubated at 70°C for 10 min., and then slowly allowed to cool to room temperature to produce dsRNA. The dsRNA products were precipitated using isoproterenol, purified on G25 columns, and quantified prior to use in experiments.

### Total RNA extraction, RT-PCR, and Real-time RT-qPCR analysis

Total RNA was prepared using TRIzol Reagent (Invitrogen) from GIV-infected GK cells at an m.o.i. of 10; total RNA was sampled at 1, 3, 6, 12, and 18 h post-infection. Before GIV infection, appropriate cultures were transfected with GIV-CARD dsRNA (10 μg for each 10 cm culture dish) or PBS (control) using LipofectAMINE 2000 (Invitrogen) in accordance with the manufacturer’s instructions. HiScript І Reverse Transcriptase (AM0670-1000, BIONAVAS) kit was used to generate cDNA with 2 μg total RNA as template, under the following conditions: 5 min. at 65°C, 30 min. at 42°C, and 15 min. at 70°C. For RT-PCR, forward and reverse primer sets (GIV-027L: GIV-CARD-5UTR-F and GIV-CARD-3UTR-R; GIV-029L, Accession Number: AAV91052: GIV-TNFR029L-F and GIV-TNFR029L-R; GIV-030L, Accession Number: AAV91053: GIV-TNFR030L-F and GIV-TNFR030L-R; GIV-065R, Accession Number: AAV91081: GIV-TNFR065R-F and GIV-TNFR065R-R; GIV-078R, Accession Number: AAV91093: GIV-Bcl-F and GIV-Bcl-R; GIV-045R, Accession Number: AAV91066: GIV-MCP-F and GIV-MCP-R) (Table 1) were used to detect GIV RNA. PCR was performed with a 50-fold diluted cDNA as template, under the following conditions: 1 cycle of 5 min. at 94°C; 35 cycles of 30 s at 94°C, 30 s at 50°C, 30 s at 72°C; followed by 5 min. at 72°C. Grouper β-actin (Primers: Grouper-β-actin-F and Grouper-β-actin-R) (Table 1) was used as an internal control. Real-time RT-qPCR was performed using a 50-fold dilution of cDNA, gene-specific primer sets (GIV-027L: qGIV-CARD-F and qGIV-CARD-R; GIV-029L: qGIV-TNFR029L-F and qGIV-TNFR029L-R; GIV-030L: qGIV-TNFR030L-F and qGIV-TNFR030L-R; GIV-065R: qGIV-TNFR065R-F and qGIV-TNFR065R-R; GIV-078R: qGIV-Bcl-F and qGIV-Bcl-R; GIV-045R: qGIV-MCP-F and qGIV-MCP-R) (Table 1), and SYBR Green PCR Master Mix (4334973, Life technologies), with a 7900HT Fast Real-Time PCR System (Applied Biosystems) running the following program: 1 cycle of 5 min. at 95°C; 45 cycles of 15 s at 95°C, 1 min. at 60°C. Grouper β-actin (Primers: Grouper-β-actin-F and Grouper-β-actin-R) (Table 1) was used as an internal control, and gene expression levels were calculated by the comparative Ct method. Three independent RT-qPCR experiments were performed (n = 3).

### Virus titration

Wells of a 48-well plate were seeded with approximately 5 × 10^4^ of transfected or non-transfected GK cells; plates were incubated overnight to allow the cells to attach. The GIV stock (1 × 10^8^ TCID_50_/ml) was diluted serially from 10^-3^ to 10^-9^ with L-15 media containing 2% FBS; 500 μl of dilutions were used to inoculate each well. After infection, the cytopathic effect (CPE) was recorded for 7 days, and the infective titers were determined from the 50% tissue culture infective dose (TCID_50_ ml^-1^) [39].

### Immunocytochemical staining

HeLa cells were transfected with pcDNA3CF_GIV-CARD using LipofectAMINE 2000 (Invitrogen). For the intrinsic pathway assay, the transfected cells were incubated for 18 h, then irradiated with UV at 0.24 J using a UV Stratalinker 1800 (Stratagene), and incubated for 9 h. For the extrinsic pathway assay, the transfected cells were incubated for 18 h, and then treated with 0.5 μg/ml Anti-Fas CH11 antibody (05–201, Millipore) and 1 μg/ml Actinomycin D (Calbiochem) for 12 h [40]. After this second incubation, the treated cells were fixed in 4% paraformaldehyde in PBS for 15 min. at room temperature. After rinsing three times with PBS, cells were permeabilized by incubation with PBST for 5 min. After rinsing the cells twice with PBS, cells were blocked by incubation with 10% bovine serum albumin in PBS (10% BSA/PBS) for 30 min. at 37°C, and then incubated with mouse monoclonal ANTI-FLAG M2 antibody (F1804, Sigma) in 3% BSA/PBS (1:500 dilution) for 2 h. The cells were rinsed three times with PBS, and then incubated with goat anti-mouse antibody-rhodamine (AP124R, Chemicon) in 3% BSA/PBS (1:1000 dilution) for 45 min. Cell nuclei were stained with DAPI (D1306, Invitrogen). The resulting samples were analyzed by fluorescence microscopy.

### TUNEL assay

Apoptotic cells were monitored by terminal deoxynucleotidyl transferase-mediated UTP end labeling (TUNEL) staining using an *in situ* cell death detection kit (Roche), according to the manufacturer’s instructions. Cells cultured on Millicell EZ slides (Millipore) were fixed in 4% paraformaldehyde for 15 min. at room temperature, washed twice with PBS, and incubated in permeabilization solution (PBST) for 5 min. After rinsing the slides twice with PBS, the area around the samples was dried and incubated with the TUNEL reaction mixture (terminal deoxynucleotidyl transferase and nucleotides) for 1 h at 37°C in the dark. The slides were rinsed three times with PBS, and then observed under a fluorescence microscope. The percentage of apoptotic cells was determined by counting the total number of cells double-positive for fluorescein and DAPI.

### Caspase activity assay

For the caspase activity assay, approximately 10^6^ HeLa cells were seeded onto a well of a six-well plate, and then transfected with 10 μg pcDNA3CF_GIV-CARD. HeLa cells transfected with pcDNA3CF were used as controls. At 18 h after transfection, cells were treated with Anti-Fas CH11 antibody (0.5 μg/ml) and Actinomycin D (1 μg/ml) for 9 h. Caspase activity assays were performed using Caspase Colorimetric Substrate Set II Plus (BioVision), in accordance with the manufacturer’s instructions. Briefly, the treated cells were resuspended in 50 μl chilled cell lysis buffer on ice for 10 min., and then centrifuged at 10,000 × *g* for 1 min. The resulting supernatant was diluted to 100 μg protein/50 μl cell lysis buffer, and added to a fresh tube containing 50 μl 2 × reaction buffer with 10 mM DTT. Five microliters of 4 mM ρNA-conjugated substrates were subsequently added to each tube, and the mixtures were incubated at 37°C for 1 h. Caspase activities were recorded at 405 nm with a microtiter plate reader (Molecular Devices, SpectraMax M5).

### Statistical analysis

The independent samples t-test (performed with SPPS ver.17) was used to determine if two sets of data were significantly different from each other. A P-value of less than 0.05 was considered to be statistically significant.

## Results

### Features of GIV-CARD

**Fig 1:**
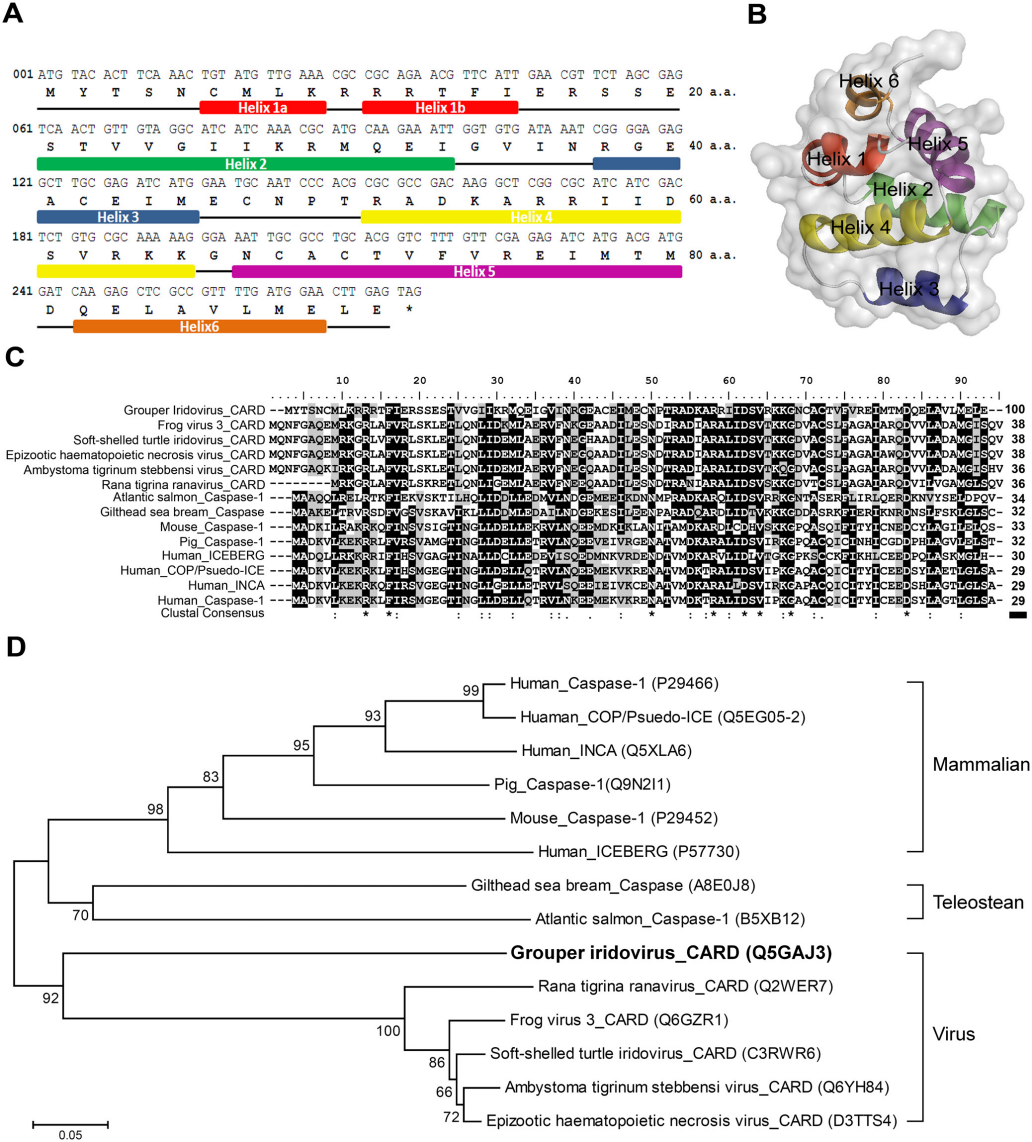
Features of grouper iridovirus caspase recruitment domain (GIV-CARD) protein. (A) Nucleotide and deduced amino acid sequences of GIV-CARD. The GIV-027L open reading frame consists of 276 nucleotides including a TAG stop codon, and is predicted to encode a CARD protein of 91 amino acids. Six helix regions are marked as bold lines underneath the amino acid sequence. (B) Predicted structure of the GIV-CARD protein. SWISS-MODEL was used to predict the tertiary structure of GIV-CARD based on the human ICEBERG protein crystal structure. The predicted α-helix regions (helix 1a and 1b (red), helix 2 (green), helix 3 (blue), helix 4 (yellow), helix 5 (purple), helix 6 (orange)) and coils (white) are shown. (C) Multiple alignments of the GIV-CARD and other virus, mammalian, and teleostean CARD-encoding genes. Fully-conserved residues are marked by an asterisk. Strongly conserved positions are marked by colons (:) and weakly conserved positions by periods (.). Numbers above the bold line in the bottom-right-hand corner indicate percent identity to GIV-CARD. (D) Phylogenetic relationship of GIV-CARD with CARD-encoding genes of mammals, teleosts, and other viruses. The Neighbor-Joining Tree was constructed using the CARD domains of caspase-1 in human, pig, and mouse, and CARD domain-only proteins from human (three proteins), two teleosts, and six viruses. Accession numbers for each sequence are given in parentheses.

In the genome of grouper iridovirus, we identified an open reading frame (ORF 027L; located from nt 32549 to 32824) encoding a CARD-only protein (GIV-CARD) of 91 amino acids with a predicted molecular weight of 10,505.42 Daltons [31]. Secondary structure modeling using SWISS-MODEL suggests that GIV-CARD contains six α-helices (Fig 1A). We proceeded to use the crystal structure of human ICEBERG (PDB ID: 1DGN) as a template [12] to predict the 3D structure of GIV-CARD with SWISS-MODEL (Fig 1B). Multiple sequence alignments of GIV-CARD revealed high identities to caspase-1 and CARD-only proteins of mammals (including human_ICEBERG, human_COP/Pseudo-ICE, and humam_INCA), teleosts, and viruses (including the CARD proteins of *Rana tigrina* ranavirus, frog virus 3, soft-shelled turtle iridovirus, *Ambystoma tigrinum* stebbensi virus, and epizootic haematopoietic necrosis virus). Relatively high identities (36% to 38%) were observed between GIV-CARD and homologous proteins in other viruses, but identities were lower when compared with proteins in teleosts and mammals, at 32% to 34% and 29% to 33%, respectively (Fig 1C). Although the alignment revealed that vertebrate and viral CARD domains were highly similar, phylogenetic tree analysis separated them into two groups; in terms of phylogeny, GIV-CARD is more closely related to CARD proteins in other viruses than those in vertebrates (Fig 1D).

### Expression of GIV-CARD in GIV-infected GK cells

**Fig 2:**
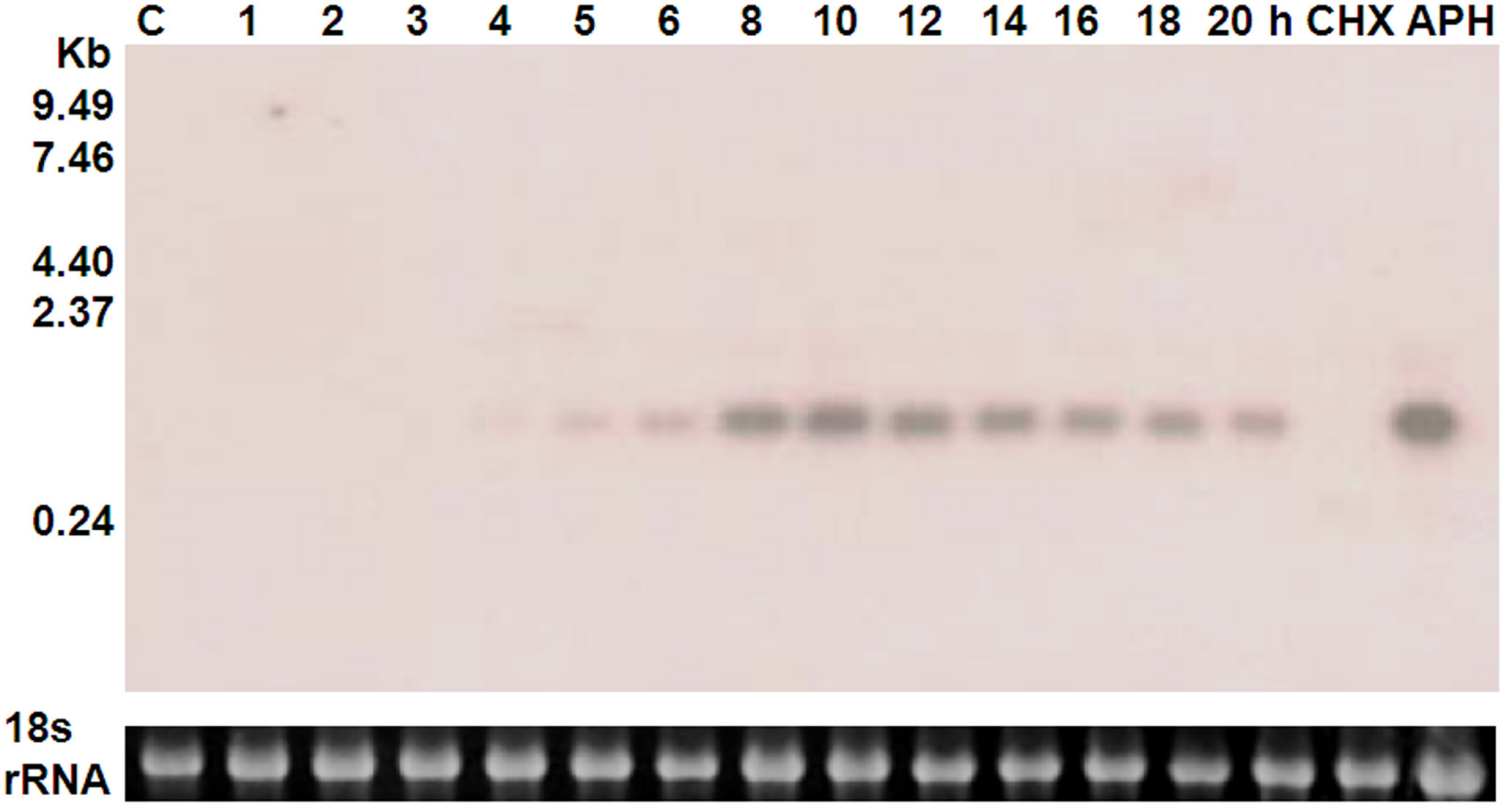
Northern blot hybridization against grouper iridovirus caspase recruitment domain (GIV-CARD) mRNA in GIV-infected GK cells. Northern blot hybridization against grouper iridovirus caspase recruitment domain (GIV-CARD) mRNA in GIV-infected GK cells at various time points after GIV infection (10 m.o.i.), or in the presence of cycloheximide (CHX) or aphidicolin (APH). Blots against 18 s rRNA are shown for each sample to represent total RNA quality and quantity. C, control, no GIV infection.

GIV inhibits apoptosis at an early stage of infection [30]. To determine whether or not the GIV-CARD gene is expressed during the early stage of GIV infection, we used total RNA extracted from GK cells infected with GIV (10 m.o.i.) at various time points to examine viral CARD expression by Northern blot. Transcripts of approximately 0.3 kb were observed. Low expression was observed as early as 4 hours post-infection (hpi), with high expression from 8 to 14 hpi (Fig 2). We proceeded to examine viral CARD gene expression in the presence of APH (a DNA polymerase inhibitor) or high concentrations of CHX, which block translation and limit viral gene expression to immediate early transcripts [41]. We observed that viral CARD expression was increased by APH, but not by CHX (Fig 2). Therefore, these results indicate that the GIV-CARD gene is an early gene, but not an immediate early gene.

### Subcellular localization of GIV-CARD

**Fig 3:**
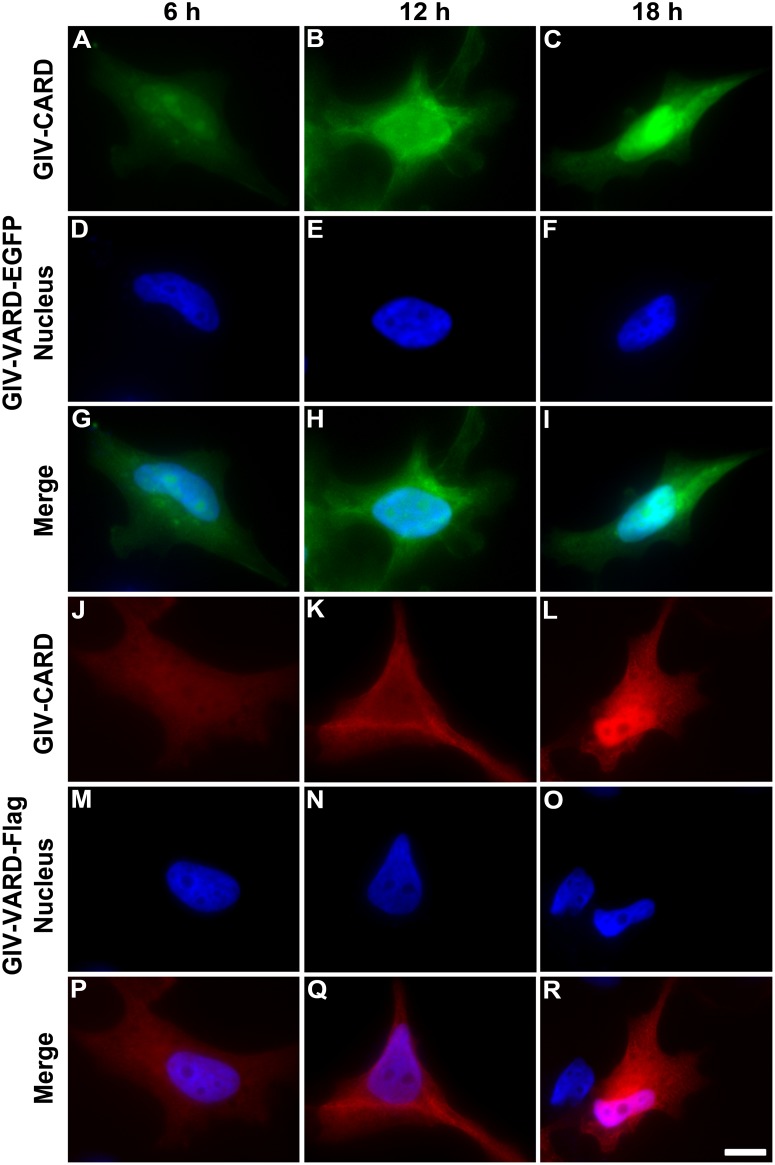
Subcellular localization of grouper iridovirus caspase recruitment domain (GIV-CARD) proteins in HeLa cells. Recombinant GIV-CARD-EGFP proteins (green fluorescence) were detected at 6 h (A), 12 h (B) and 18 h (C). DAPI (blue fluorescence) was used to stain nuclei (D, E, and F). Panels G, H, and I are merged images of panels A and D, panels B and E, and panels C and F, respectively. GIV-CARD-FLAG recombinant proteins (red fluorescence) were detected by immunocytochemical staining and shown for the indicated times (J, K, and L). DAPI (blue fluorescence) was used to stain nuclei (M, N, and O). Panels P, Q, and R are merged images of panels J and M, panels K and N, and panels L and O, respectively. Scale bar = 10 μm.

To examine the subcellular localization of GIV-CARD protein, we transiently transfected HeLa cells with one of two expression vectors, pEGFP-N1_GIV-CARD or pcDNA3CF_GIV-CARD, and examined expression by direct fluorescence observation or immunofluorescence staining. As early as 3 h post-transfection (p.t.), low signals for both GIV-CARD-EGFP and GIV-CARD-FLAG recombinant protein were detected (data not shown). Expression of GIV-CARD-EGFP was clearly observed in the cytoplasm at 6 h p.t. (Fig 3A). A few cells with clear fluorescence around the nucleus could be observed at 12 h p.t. (Fig 3B). However, the majority of fluorescence was detected in the nucleus at 18 h p.t. (Fig 3C). The transfected cells were co-stained with DAPI to reveal the cell nucleus (Figs 3D–3F; 3M–3O). To better understand the process by which GIV-CARD-EGFP translocates into the nucleus, time-lapse recordings were made (S1 File). Similar results were also obtained upon immunofluorescence staining of the GIV-CARD-FLAG recombinant protein (Fig 3J–3R). These observations suggest that GIV-CARD recombinant proteins are expressed in the cytoplasm, and ultimately migrate and translocate into the nucleus.

### Inhibition of GIV gene expression and virus infection by knockdown of GIV-CARD in GK cells

**Fig 4:**
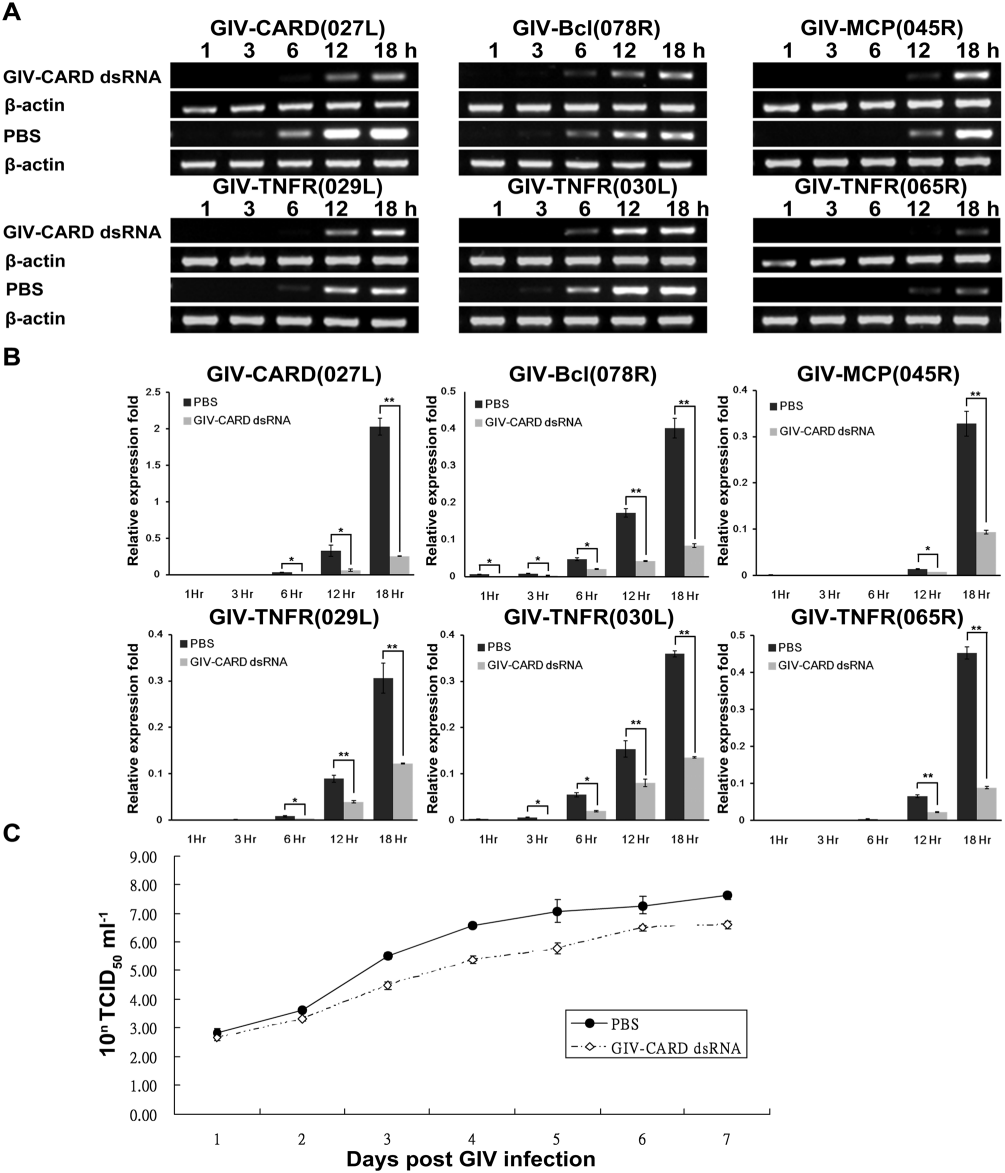
Transfection of GK cells with grouper iridovirus caspase recruitment domain (GIV-CARD) dsRNA inhibits expression of six GIV genes, and reduces GIV infection. Expression of six GIV genes after GIV infection was decreased in GIV-CARD dsRNA transfected-GK cells as compared to control cells. RT-PCR (A) and real-time RT-qPCR (B) were used to determine gene expression level. The expression level of β-actin was used as an internal template control. PBS was used as a transfection control. Data represent the mean ± S.D. (n = 3); * P<0.05, ** P<0.01 as compared to the control group. (C) Viral titer following GIV infection was reduced in GK cells transfected with GIV-CARD dsRNA as compared to controls. Data are shown as means ± S.D. (n = 3).

To evaluate the effect of GIV-CARD on the expression of viral genes in GK cells infected with GIV, GK cells were transfected with GIV-CARD dsRNA (generated *in vitro*) prior to infection. The GIV-CARD dsRNA was expected to be processed into smaller GIV-CARD siRNA fragments through the RNA interference mechanism in GK cells. The transcripts of GIV-CARD and five viral genes, including two immediate early genes (GIV-Bcl 078R and GIV-TNFR 030L), two early genes (GIV-TNFR 029L and GIV-TNFR 065R), and one late gene (GIV-MCP 045R), were analyzed by RT-PCR and real-time qPCR. RT-PCR revealed that the expression of all six transcripts was significantly reduced by knockdown of GIV-CARD in GK cells during viral infection (Fig 4A). To better quantify the inhibition of viral gene transcription by knockdown of GIV-CARD in GIV-infected GK cells, we performed real-time qPCR. Levels of all six viral genes at 1 and 3 hpi were difficult to quantify. Expression of viral immediate early genes significantly increased from 6 hpi, whereas expression of early and late genes increased from 12 and 18 hpi, respectively. Between 12 and 18 hpi, expression of GIV-CARD, GIV-Bcl, GIV-MCP, GIV-TNFR(029L), GIV-TNFR(030L), and GIV-TNFR(065R) decreased by 81–87, 76–79, 43–72, 56–60, 47–62, and 66–80%, respectively, as compared to the PBS control (Fig 4B). Furthermore, transfection of GK cells with GIV-CARD dsRNA severely reduced the cytopathic effect of GIV infection, and resulted in a 20-fold reduction in virus yield (Fig 4C). The inhibition of (i) virus infection and (ii) GIV gene expression in GIV-CARD knock-down GK cells implies that GIV-CARD plays an important role in GIV infection.

### GIV-CARD inhibits apoptosis in HeLa cells

**Fig 5:**
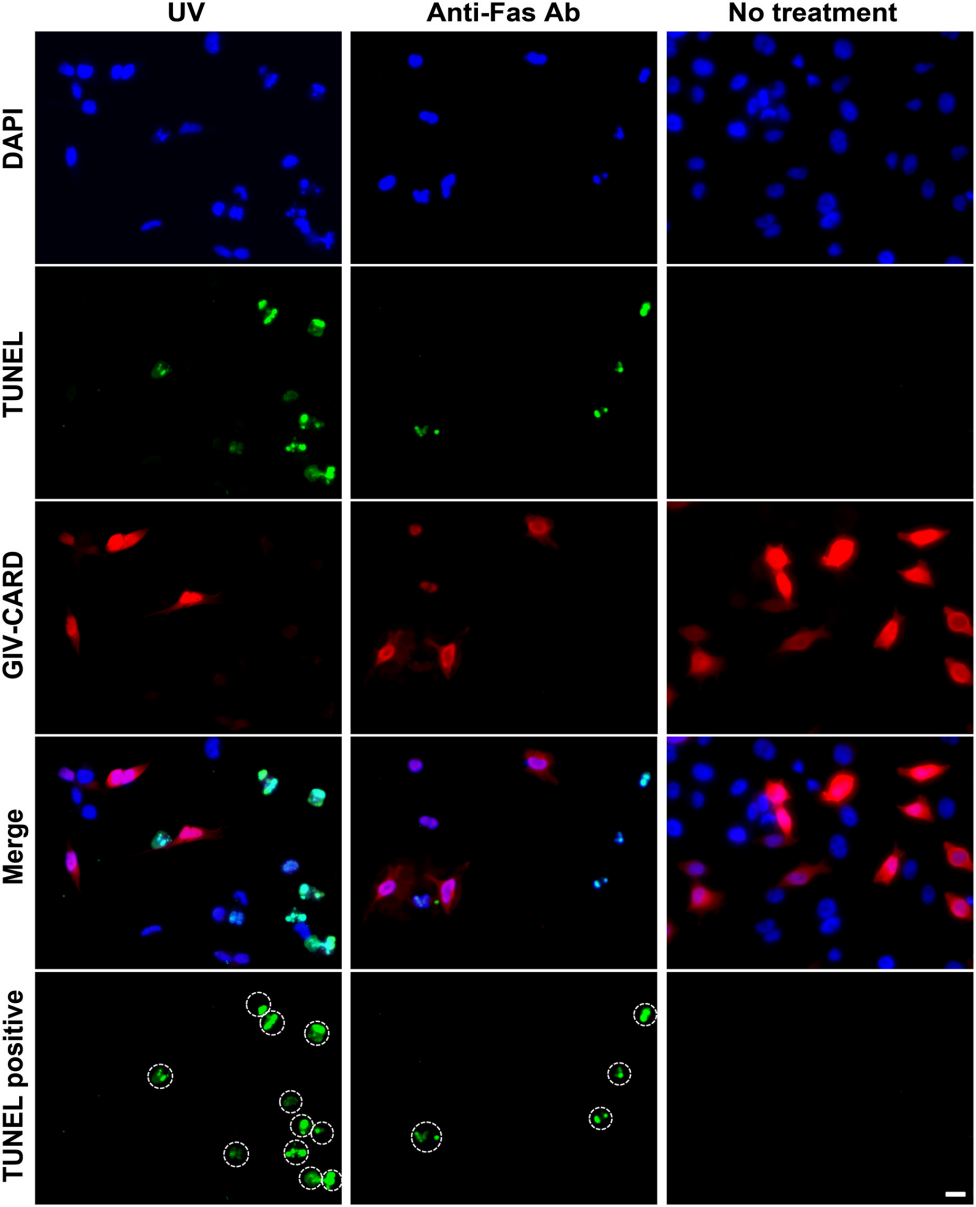
Grouper iridovirus caspase recruitment domain (GIV-CARD) protein protects HeLa cells from UV- or anti-Fas antibody-induced apoptosis. Immunocytochemistry of HeLa cells transfected with pcDNA3CF_GIV-CARD and irradiated with 0.24 Joules UV or treated with 0.5 μg/ml anti-Fas CH11 antibody. Nuclei (blue), apoptotic bodies (green), and GIV-CARD-expressing cells (red) were detected by DAPI staining, TUNEL assay, and immunocytochemical staining, respectively. TUNEL positive: indicating TUNEL positive cells. Scale bar = 20 μm.

To investigate whether GIV-CARD inhibits the intrinsic apoptosis pathway, we irradiated pcDNA3CF_GIV-CARD transfected-HeLa cells with UV, and then performed simultaneous immunocytostaining against nuclei, apoptotic cells, and GIV-CARD. After UV treatment, HeLa cells without GIV-CARD expression underwent apoptosis, as detected using the TUNEL assay (S1 Fig and Fig 5). However, no obvious apoptotic signals were observed in GIV-CARD-expressing cells (Fig 5). To determine the efficiency of the anti-apoptotic ability of GIV-CARD, we calculated the percentage of apoptosis inhibition from the total number of apoptotic GIV-CARD-expressing cells. We observed that apoptosis was inhibited in up to 73.9% of cells expressing high levels of GIV-CARD, whereas apoptosis was inhibited in 52.4% of cells expressing low levels of GIV-CARD (Table 2). Next, we proceeded to determine if GIV-CARD inhibits the extrinsic apoptotic pathway. HeLa cells were transfected with pcDNA3CF_GIV-CARD and treated with anti-Fas antibody; the latter mimics the Fas death ligand, thereby triggering the Fas death receptor apoptosis pathway. As observed in the previous experiment, HeLa cells without GIV-CARD expression underwent apoptosis (S1 Fig and Fig 5). In contrast, no obvious apoptotic signals were observed in the GIV-CARD-expressing cells (Fig 5). We found that apoptosis was inhibited by up to 70.0% in cells expressing high levels of GIV-CARD, but only by 37.9% in cells expressing low levels of GIV-CARD (Table 2).

**Table 2:** Inhibition of UV- or anti-Fas antibody-triggered apoptosis by GIV-CARD protein as determined through immunocytochemistry analysis.

	Total number of apoptotic cells in GIV-CARD expressing cells (%)			Percentage of apoptosis inhibition		
**GIV-CARD expression level**	**-**	**+**	**++**	**-**	**+**	**++**
**0.24 J UV**	**484/818 (59.1)**	**64/227 (28.1)**	**24/155 (15.4)**	**0**	**52.4**	**73.9**
**0.5 μg/ml Anti-Fas Ab**	**677/886 (76.4)**	**73/169 (47.4)**	**22/96 (22.9)**	**0**	**37.9**	**70.0**
**No treatment**	**6/305 (1.9)**	**4/255 (1.5)**	**1/54 (1.8)**	**0**	**21.0**	**5.2**

### Inhibition of caspase-8 and -9 activities by recombinant GIV-CARD

**Fig 6:**
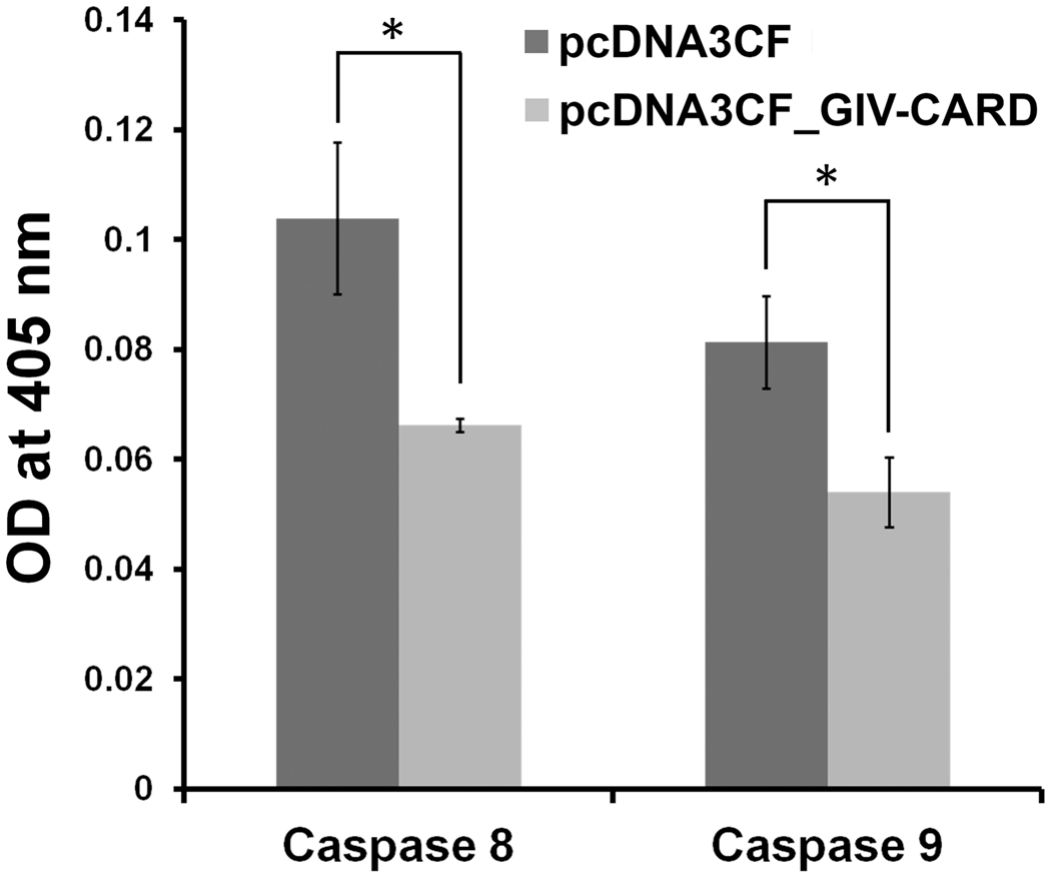
Inhibition of caspase activity in HeLa cells over-expressing grouper iridovirus caspase recruitment domain (GIV-CARD) protein. Capase-8 and -9 exhibited reduced activity in cells expressing GIV-CARD following anti-Fas antibody-induced apoptosis. Data represent the mean ± S.D. (n = 3); * P<0.05 as compared to the control group.

The two primary apoptosis signaling pathways, intrinsic and extrinsic, activate the initiator caspases, caspase-9 and caspase-8, respectively. Therefore, it is important to understand the effect of GIV-CARD on the activities of these caspases. To address this issue, we examined the activities of caspase-8 and -9 in GIV-CARD-expressing HeLa cells after treatment with anti-Fas antibody. Compared with vector control, the activities of caspase-8 and -9 were significantly (P<0.05) reduced in GIV-CARD-expressing cells by 36% and 34%, respectively (Fig 6). These results indicate that GIV-CARD functions as an anti-apoptotic protein to provide an environment for virus propagation.

## Discussion

Viruses have developed several strategies to overcome host defenses, in order to create a suitable environment for viral propagation. Apoptosis is an efficient means of eliminating virus-infected cells from the host, and therefore viruses have acquired certain host genes to counteract apoptotic pathways. We previously reported that grouper iridovirus can suppress UV-induced apoptosis at an early stage of viral infection, and that the virus encodes Bcl-like protein (078R), which appears on mitochondrial membranes to block the apoptotic pathway [30]. In the present study, we observed that the virally-encoded CARD-only protein (027L) translocates from the cytoplasm to the nucleus, and can inhibit both the mitochondrial and death receptor apoptosis pathways. To our knowledge, GIV-CARD is the first viral CARD to be shown to be an anti-apoptotic protein.

Although no obvious nuclear localization sequence (NLS) was found in the GIV-CARD protein, recombinant GIV-CARD was observed to accumulate in the nucleus. Nuclear localization of IкBα, a postinductional repressor of NF-кB/Rel proteins, is mediated by a novel nuclear import sequence within the second ankyrin repeat [42]. Therefore, it is possible that GIV-CARD also possesses a currently unknown nuclear import sequence. Another possibility is that GIV-CARD functions as a decoy which associates with target protein(s) through CARD-CARD interactions, and then accompanies its target into the nucleus. It has been hypothesized that the transmission of the apoptotic signal from plasma membrane receptors to effector caspases requires the association of proteins with homologous domains of the same class, such as DD, DED, or CARD. Based on secondary structure analysis, DD, DED, and CARD were all predicted to consist of six α-helices; these three interaction domains are therefore hypothesized to have evolved from a common ancestor [1]. Thus, if GIV-CARD acts as a decoy, it may recruit the protein containing the most similar CARD. Multiple protein sequence alignments showed that GIV-CARD had the greatest identity with CARDs of other *Ranavirus* species (36% to 38% identity), implying that these viral CARDs evolved from a common ancestor. Although GIV-CARD exhibits less identity with mammalian than viral host teleostean CARDs (Fig 1C), GIV-CARD-expressing human HeLa cells presented with reduced caspase activity (Fig 6); this suggests that viral CARD may be an effective decoy for targeting proteins involved in apoptotic signaling. One possible target of GIV-CARD is caspase-8, which translocates into the nucleus of apoptotic neurons [43]; however, this hypothesis requires further investigation. Of interest, we performed co-immunoprecipitation experiments to identify eight potential GIV-CARD-binding proteins, with molecular weights from 50 to 10 kDa (S2 Fig).

Upon viral infection, host intracellular inflammasomes sense the double stranded DNA derived from DNA viruses, and stimulate the activation of caspase-1 through the recruitment of procaspase-1 by CARD-containing proteins. In turn, the activated caspase-1 induces the maturation and secretion of inflammatory cytokines, such as IL-1β and IL-18, to initiate innate immune defenses [44]. Inflammasome-dependent caspase-1 activity can result in a highly inflammatory form of cell death, known as pyroptosis, in myeloid cells [45]. To avoid excessive inflammasome activation, caspase-1-dependent pyroptosis can be inhibited by CARD-only proteins (human ICEBERG, COP/Pseudo-ICE, and INCA). Previous studies have indicated that ICEBERG and COP/Pseudo-ICE serve as non-enzymatic decoys that regulate caspase-1 activity [12, 14]. INCA physically interacts with procaspase-1 and blocks the release of mature IL-1β from LPS-stimulated macrophages, but does not induce the NF-кB activation pathway [13]. ICEBERG, COP/Pseudo-ICE, and INCA may compete with other CARD proteins for binding to bipartite-CARDs or multi-domain CARDs, and in this way, may act as inhibitors of either caspase or NF-кB activation pathways [11]. To our surprise, GIV-CARD also exhibits relatively high identity with these human CARD-only proteins (29%-30%) and CARD of mammalian caspase-1 (29%-33%). We hypothesize that viral GIV-CARD may mimic human CARD-only anti-inflammasome proteins to compete with procaspase-1 binding to the inflammasome, thereby disrupting host inflammasome-dependent innate and adaptive immunity; future studies may investigate this proposal.

Viral genes expressed during infection can be divided into three classes: immediate early genes, early or delayed-early genes, and late genes [46]. Upon infection, viruses express immediate early and early genes, and then replicate viral DNA; the virus subsequently expresses late structural genes, allowing it to assemble the virion particle. Infected cells initiate apoptosis to interrupt viral propagation; however, viruses seek to prevent this process in order to maximize the production of progeny. To this end, GIV has acquired several potential anti-apoptotic proteins, including TNFR (029L), TNFR (030L), TNFR (065R), Bcl (078R), and CARD (027L). Bcl (078R) [30] and TNFR (030L) (data not shown) are immediate early genes, and may serve as the first arm of the apoptosis inhibition system at the mitochondrial and plasma membranes, respectively. We observed expression of GIV-CARD mRNA as early as 4 h post-infection, which is just 2 h later than that of the immediate early gene GIV-Bcl [30]; however, GIV-CARD is not expressed in the presence of CHX, and therefore its transcription requires newly synthesized proteins. Thus, GIV-CARD may act as part of a secondary apoptosis inhibition system. However, the subcellular localization of GIV-CARD, and its effect on the core enzymes of apoptotic signaling (initiator caspases), underscores its potential importance in ensuring apoptosis inhibition if the primary apoptosis inhibition systems should fail, or in otherwise maintaining such inhibition.

## Supporting Information

S1 Fig UV or anti-Fas antibody induces apoptosis in HeLa cells. Immunocytochemistry of HeLa cells irradiated with 0.24 Joules UV or treated with 0.5 μg/ml anti-Fas CH11 antibody. Nuclei (blue) and apoptotic bodies (green) were detected by DAPI staining and TUNEL assay, respectively. Scale bar = 40 μm.
(TIF)

S2 Fig Immunoprecipitation of GIV-CARD-binding proteins from HeLa cells expressing GIV-CARD-FLAG. GIV-CARD-binding proteins were resolved by 12% SDS-PAGE and developed with silver stain. M: molecular weight markers. Lane 1: anti-Flag antibody-conjugated agarose beads (antibody control). Lane 2: lysate from HeLa cells transfected with pcDNA3CF (vector control), precipitated with anti-Flag antibody-conjugated agarose beads. Lane 3: lysate from HeLa cells transfected with pcDNA3CF_GIV-CARD, precipitated with anti-Flag antibody-conjugated agarose beads. Lane 4: lysate from HeLa cells transfected with pcDNA3CF_GIV-CARD and subjected to irradiation with 0.24 J UV, precipitated with anti-Flag antibody-conjugated agarose beads. Arrows indicate the potential GIV-CARD-binding proteins. The presence of GIV-CARD-FLAG was confirmed using anti-Flag antibody.
(TIF)

S1 File Nuclear translocation of GIV-CARD-EGFP in HeLa cells. HeLa cells were cultured in DMEM media supplemented with 10% FBS in a 3.5 cm culture dish (Nunc) at 37°C overnight. Cells were transfected with 1 μg pEGFP-N1_GIV-CARD plasmid DNA using LipofectAMINE 2000 (Invitrogen), in accordance with the manufacturer’s instructions. Three hours after transfection, time-lapse images of GIV-CARD-EGFP-expressing cells were captured every 20 min. using a fluorescence microscope system (Axiovert 200M Zeiss/Photometrics CoolSnap HQ). Images were merged to form an animation using Metamorph software. The file includes images from 6 h to 18 h post-transfection.
(AVI)
