# Supplementary figures and images for: Iridovirus CARD Protein Inhibits Apoptosis through Intrinsic and Extrinsic Pathways
Source: PLoS One. 2015 Jun 5;10(6):e0129071. doi: 10.1371/journal.pone.0129071 (PMC4457926; doi:10.1371/journal.pone.0129071)

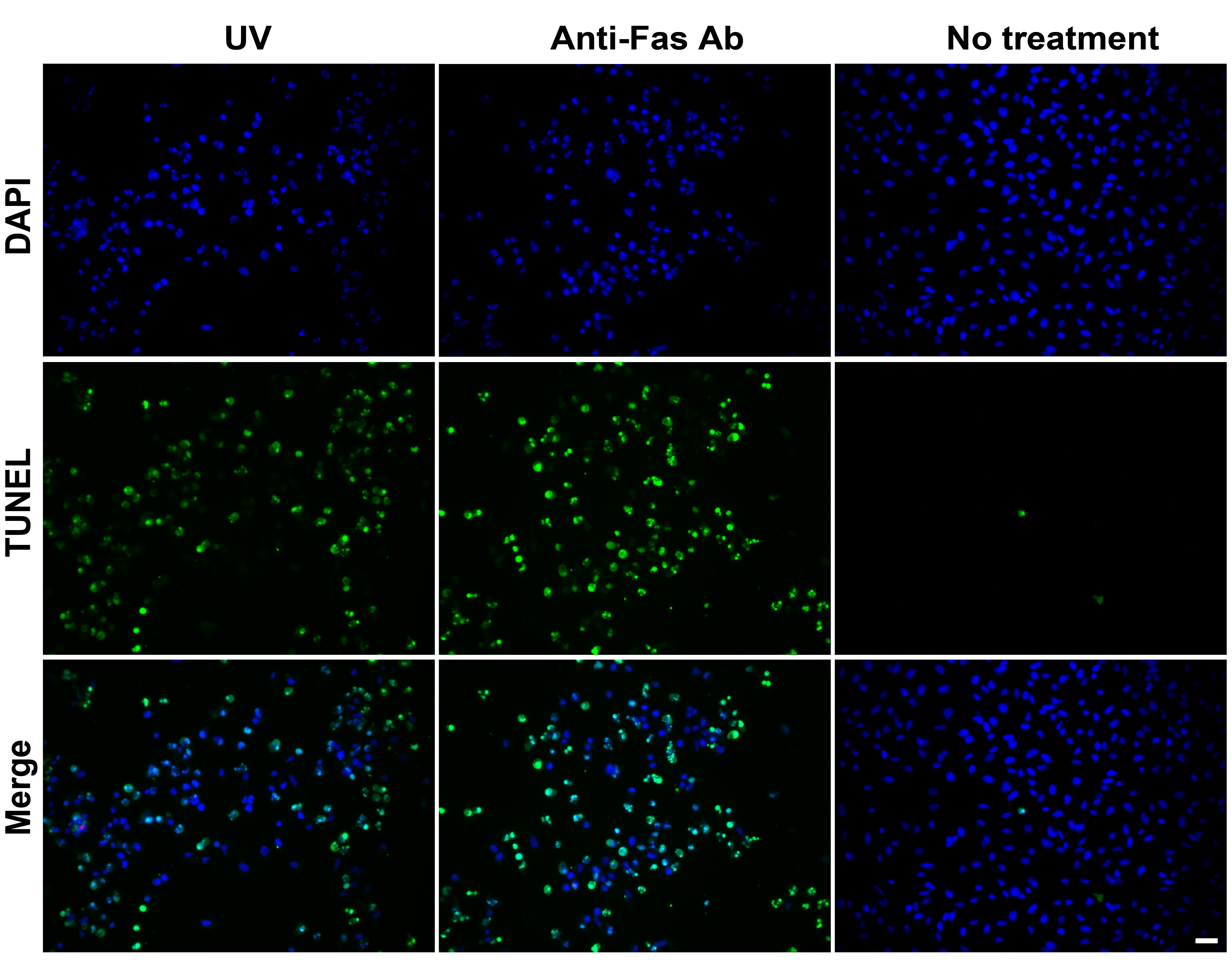

Supplement: S1 Fig — Immunocytochemistry of HeLa cells irradiated with 0.24 Joules UV or treated with 0.5 μg/ml anti-Fas CH11 antibody. Nuclei (blue) and apoptotic bodies (green) were detected by DAPI staining and TUNEL assay, respectively. Scale bar = 40 μm. (TIF) [file pone.0129071.s001.tif]

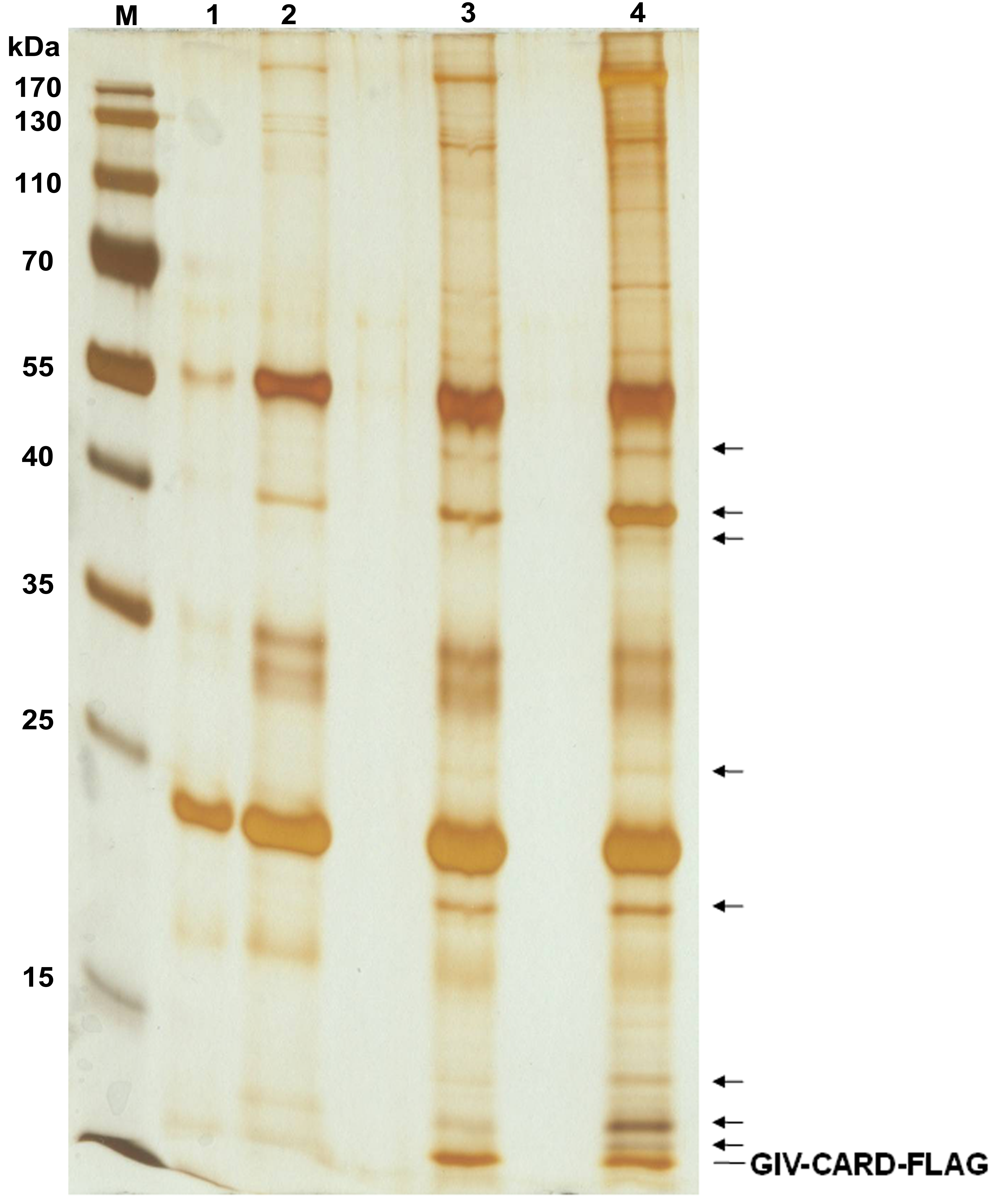

Supplement: S2 Fig — GIV-CARD-binding proteins were resolved by 12% SDS-PAGE and developed with silver stain. M: molecular weight markers. Lane 1: anti-Flag antibody-conjugated agarose beads (antibody control). Lane 2: lysate from HeLa cells transfected with pcDNA3CF (vector control), precipitated with anti-Flag antibody-conjugated agarose beads. Lane 3: lysate from HeLa cells transfected with pcDNA3CF_GIV-CARD, precipitated with anti-Flag antibody-conjugated agarose beads. Lane 4: lysate from HeLa cells transfected with pcDNA3CF_GIV-CARD and subjected to irradiation with 0.24 J UV, precipitated with anti-Flag antibody-conjugated agarose beads. Arrows indicate the potential GIV-CARD-binding proteins. The presence of GIV-CARD-FLAG was confirmed using anti-Flag antibody. (TIF) [file pone.0129071.s002.tif]
